# Onions

**Published:** 1893-05

**Authors:** 


### ONIONS.

Onions are one of the most nutritious, healthful and detestable articles of food found in our markets.

A few grains of roasted coffee eaten immediately afterwards, or a teaspoonful or two of vinegar swallowed, removes at once the odor from the breath. If onions are half boiled, the water thrown away, and are then put in soup to be boiled " done," the odor will be little noticed.

